# Utility of bile acids in large airway bronchial wash versus bronchoalveolar lavage as biomarkers of microaspiration in lung transplant recipients: a retrospective cohort study

**DOI:** 10.1186/s12931-022-02131-5

**Published:** 2022-08-26

**Authors:** Chen Yang Kevin Zhang, Musawir Ahmed, Ella Huszti, Liran Levy, Sarah E. Hunter, Kristen M. Boonstra, Sajad Moshkelgosha, Andrew T. Sage, Sassan Azad, Rasheed Ghany, Jonathan C. Yeung, Oscar M. Crespin, Lianne G. Singer, Shaf Keshavjee, Tereza Martinu

**Affiliations:** 1 Toronto Lung Transplant Program, University Health Network, Toronto, Canada; 2 Biostatistics Research Unit, University Health Network, Toronto, Canada; 3 Division of General Surgery, University Health Network, Toronto, Canada; 4 Division of Respirology, Department of Medicine, University of Toronto, Toronto, ON Canada

**Keywords:** Bile acids, Biomarkers, Bronchoalveolar lavage, Large airway bronchial wash, Lung transplantation, Microaspiration

## Abstract

**Background:**

Bronchoalveolar lavage (BAL) is a key tool in respiratory medicine for sampling the distal airways. BAL bile acids are putative biomarkers of pulmonary microaspiration, which is associated with poor outcomes after lung transplantation. Compared to BAL, large airway bronchial wash (LABW) samples the tracheobronchial space where bile acids may be measurable at more clinically relevant levels. We assessed whether LABW bile acids, compared to BAL bile acids, are more strongly associated with poor clinical outcomes in lung transplant recipients.

**Methods:**

Concurrently obtained BAL and LABW at 3 months post-transplant from a retrospective cohort of 61 lung transplant recipients were analyzed for taurocholic acid (TCA), glycocholic acid (GCA), and cholic acid by mass spectrometry and 10 inflammatory proteins by multiplex immunoassay. Associations between bile acids with inflammatory proteins and acute lung allograft dysfunction were assessed using Spearman correlation and logistic regression, respectively. Time to chronic lung allograft dysfunction and death were evaluated using multivariable Cox proportional hazards and Kaplan–Meier methods.

**Results:**

Most bile acids and inflammatory proteins were higher in LABW than in BAL. LABW bile acids correlated with inflammatory proteins within and between sample type. LABW TCA and GCA were associated with acute lung allograft dysfunction (OR = 1.368; 95%CI = 1.036–1.806; *P* = 0.027, OR = 1.064; 95%CI = 1.009–1.122; *P* = 0.022, respectively). No bile acids were associated with chronic lung allograft dysfunction. Adjusted for risk factors, LABW TCA and GCA predicted death (HR = 1.513; 95%CI = 1.014–2.256; *P* = 0.042, HR = 1.597; 95%CI = 1.078–2.366; *P* = 0.020, respectively). Patients with LABW TCA in the highest tertile had worse survival compared to all others.

**Conclusions:**

LABW bile acids are more strongly associated than BAL bile acids with inflammation, acute lung allograft dysfunction, and death in lung transplant recipients. Collection of LABW may be useful in the evaluation of microaspiration in lung transplantation and other respiratory diseases.

**Supplementary Information:**

The online version contains supplementary material available at 10.1186/s12931-022-02131-5.

## Background

Bronchoalveolar lavage (BAL) is a well-established tool for minimally invasive sampling of the microenvironment in the lower airways. It is performed by wedging the tip of a flexible bronchoscope within a selected bronchopulmonary segment, instilling a volume of sterile isotonic saline sufficient to reach the alveolar space, followed by suctioning of the fluid [1, 2]. Analysis of BAL samples through biochemical, cytological, and microbiological techniques play a predominant role in the diagnosis of a wide variety of diseases in respiratory medicine. In contrast to BAL, a bronchoscopic fluid sample that has been utilized less commonly in the clinical setting is the bronchial wash, which has been defined as a non-wedged or lower volume bronchoscopic sampling where the instilled fluid does not reach the alveolar space [2]. Specifically, the large airway bronchial wash (LABW) is obtained with the bronchoscope tip in a mainstem or lobar airway. The diagnostic value of bronchial wash and BAL has been directly compared in only a few disease states, including pulmonary tuberculosis [3], lymphangitic carcinomatosis [4], and peripheral lung cancer [5]. All of these studies found that BAL had higher sensitivity.

LABW may be more advantageous than BAL as a diagnostic sample to detect pulmonary microaspiration driven by gastroesophageal reflux disease (GERD). In BAL, the presence of biochemical compounds originating from the stomach, such as pepsin and bile acids, have been shown as indicators of microaspiration in patients receiving mechanical ventilation [6], advanced lung disease patients [7–9], and lung transplant recipients [10, 11]. Given the relative proximity of the large airways and tracheobronchial space to the gastrointestinal tract, LABW may yield biomarkers of microaspiration at more clinically useful levels than BAL.

Microaspiration biomarkers with diagnostic and prognostic value are highly sought after in clinical lung transplantation as GERD-driven microaspiration has been linked to acute rejection [12], acute decline in pulmonary function [13], and development of chronic lung allograft dysfunction (CLAD) [10], presumably through inflammation and fibrosis [14]. Although early anti-reflux surgery in lung transplant recipients may slow lung function deterioration, its related risks and complications necessitate improved patient selection [15]. Our group recently reported that a specific bile acid, taurocholic acid (TCA), in BAL at 3 months post-transplant was associated with concurrent objective evidence of GERD, inflammation, and acute lung allograft dysfunction (ALAD) [16]. Moreover, BAL TCA was reduced following anti-reflux surgery [16]. Urso et al. recently showed that elevated bile acids in LABW at 3 months post-transplant were independent predictors of CLAD, death, and bacterial infections [17]. Similarly, Nakajima et al. observed that bronchial wash from donor lungs declined for implantation in recipients due to aspiration or infection had higher bile acids compared to those from accepted donor lungs. [18]

A direct comparison of the diagnostic and prognostic value of LABW and BAL bile acids in the context of microaspiration in lung transplant recipients would be timely and helpful in guiding future biomarker research. Leveraging the existing cohort from our previous BAL bile acid study [16] and our institutional protocol of routinely collecting both LABW and BAL, we aimed to compare bile acid levels between sample types and their associations with relevant short- and long-term clinical outcomes. We hypothesized that LABW bile acid at 3 months post-transplant are more strongly associated with inflammation, ALAD, CLAD, and death compared to BAL bile acid.

## Methods

### Patient selection

**Fig. 1:**
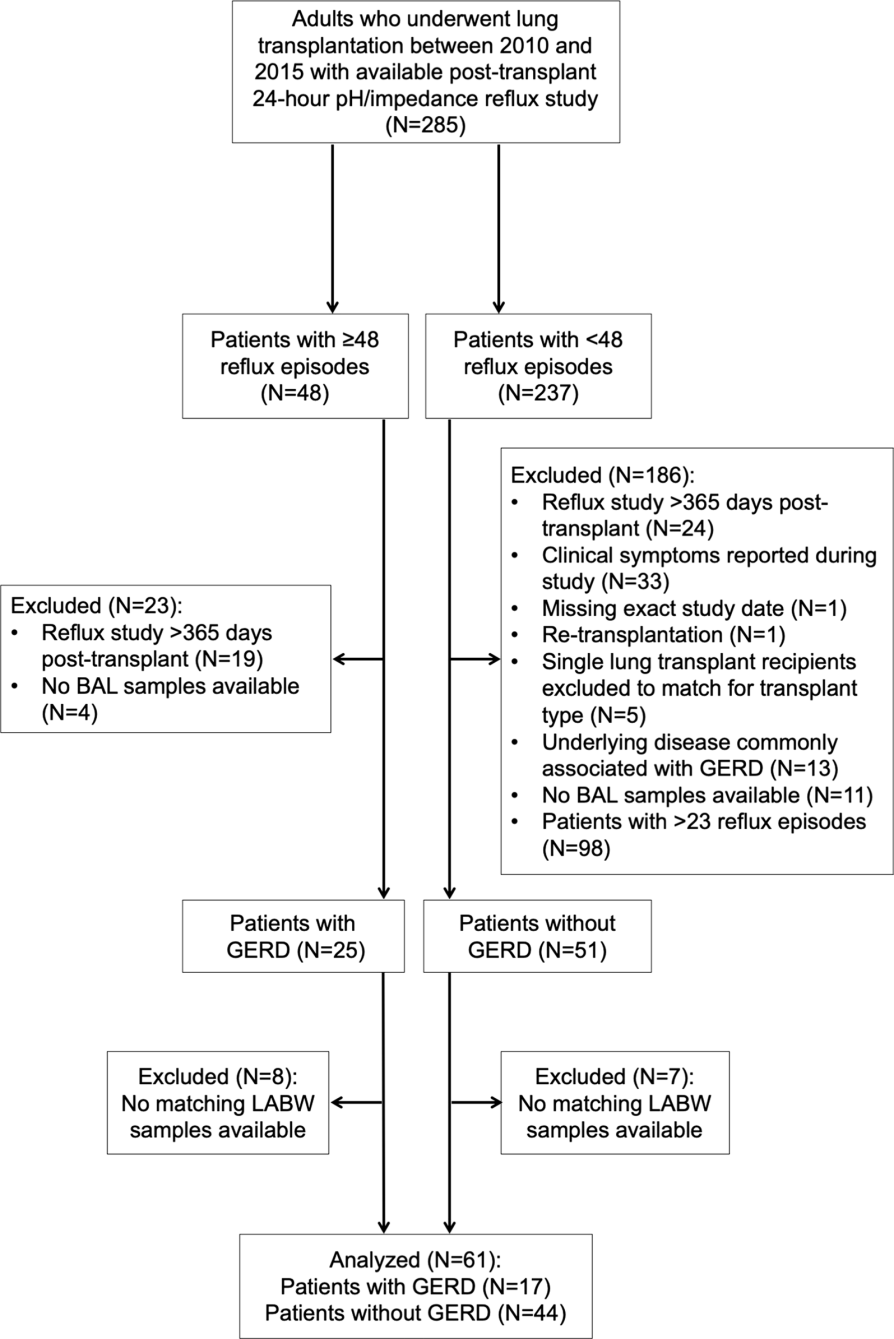
Study flow diagram. The study cohort was derived from a parent cohort of patients with or without GERD, which we previously described. We excluded 8 patients with GERD and 7 patients without GERD who did not have matching LABW samples available at 3 months after transplantation. We then combined the remaining 17 patients with GERD and 44 patients without GERD to form the study cohort of 61 patients

This single-center, retrospective cohort study was approved by the University Health Network Research Ethics Board (REB# 15-9698). Informed written consent was obtained from all patients for publication of the study data. The study cohort was derived from a previously described cohort (Fig. 1) [16]. 285 adults who underwent lung transplantation at Toronto General Hospital between 2010 and 2015 with available post-transplant 24-h esophageal pH/impedance reflux study were assessed for eligibility. Patients were categorized as with GERD (≥ 48 total reflux episodes) or without GERD (< 48 total reflux episodes) [19]. Both acidic and non-acidic episodes were included in the total number of reflux episodes as 92% of patients were on proton pump inhibitors. Patients with GERD were excluded if reflux study was performed > 365 days post-transplant or no BAL samples were available. Patients without GERD were matched 2:1 to patients with GERD by transplant type, starting from patients with the lowest number of reflux episodes, and excluded if reflux study was performed > 365 days post-transplant, clinical symptoms were reported during the study, no BAL samples were available, or other reasons detailed in Fig. 1. 8 patients with GERD and 7 patients without GERD were excluded due to no matching LABW samples available, arriving at the final study size of 61 patients (17 with GERD and 44 without GERD). Reflux testing was performed at a median of 11 days from bronchoscopy and BAL/LABW sampling.

### Sample collection, processing, and analysis

During bronchoscopy, LABW samples were collected by instilling and suctioning 20 mL of isotonic saline through a flexible bronchoscope in the mainstem bronchus. Suctioning proximal to the vocal cords was avoided. The bronchoscope was then wedged in a bronchopulmonary segment, and two BAL fractions were obtained by sequentially instilling and suctioning two 50 ml aliquots of isotonic saline. The second BAL fraction was used for this study, as it may preferentially sample the distal bronchoalveolar space [20]. The default locations of LABW and BAL were the right mainstem bronchus and right middle lobe, respectively, unless there were localizing clinical findings, such as consolidation on imaging or localized secretions on airway exam. Samples remained on ice until centrifugation for 20 min at 3184G at 4 °C; supernatants were then separated and stored at − 80 °C until analysis. Liquid chromatography with tandem mass spectrometry was used to measure levels of taurocholic acid (TCA), glycocholic acid (GCA), and cholic acid (CA). Ten inflammatory proteins (IL-1α, IL-1β, IL-6, IL-8, IL-12p70, CCL2, CCL5, defensins S100A8 and S100A12, and soluble RAGE) were measured using multiplex immunoassay (R&D Systems, USA). Club cell secretory protein (CCSP), a marker of epithelial secretory function and injury [21], was measured by ELISA (R&D Systems, USA). Analyte concentrations were not normalized since there is no universally accepted method and normalization can have unpredictable effects on results. [2]

### Clinical protocols and definitions

Standard of care was described previously by the Toronto Lung Transplant Program [22]. Briefly, patients underwent routine surveillance bronchoscopy to obtain LABW, BAL, and transbronchial biopsies at 0.5, 1.5, 3, 6, 9, 12, 18, and 24 months post-transplant. For this study, 3-month LABW and BAL samples were analyzed in order to optimize the proximity to reflux testing, which typically occurred around 3 months post-transplant as per institutional protocol. Routine pulmonary function tests were performed weekly in the first 3 months, monthly from 3 months to 2 years post-transplant, and every 3 months thereafter. Additional bronchoscopies and pulmonary function tests were performed as clinically indicated.

The most recent forced expiratory volume in 1 second (FEV1) measurement preceding the time of sample collection was used to define concurrent lung function. ALAD was defined as a ≥ 10% decline in most recent measured FEV1 at the time of bronchoscopy compared to the maximum of two preceding FEV1 measurements, consistent with our previous study [16]. Baseline lung allograft dysfunction (BLAD) was defined as failure to achieve ≥ 80% predicted FEV1 [23]. CLAD was defined in accordance to the 2019 International Society for Heart and Lung Transplantation consensus report [24]. CLAD and death outcomes were censored on February 28, 2019. No patients were lost to follow-up.

### Statistical approach

All statistical analyses were performed using R version 3.6.2. Wilcoxon signed-rank test was used to compare biomarker levels between LABW and BAL. Spearman correlation was used to compare biomarker levels in concurrent samples. Univariable logistic regression was used to assess the association between bile acid levels and ALAD or BLAD. Multivariable Cox proportional hazards models were used to determine the association of bile acid levels (as continuous variables) with time to CLAD or death. CLAD-free survival and overall survival were adjusted for major known risk factors [25]: recipient age, sex, primary disease, cytomegalovirus (CMV) mismatch, and acute rejection at time of bronchoscopy. Kaplan–Meier method was used to compare overall survival stratified by bile acid tertiles. Correction for multiple comparison was not applied due to multicollinearity of bile acids and proteins. Unadjusted *P* values are reported; a threshold of *P* < 0.05 was considered statistically significant.

## Results

### Study population

Sixty-one patients were included in the study (Fig. 1). Baseline patient characteristics are described in Table 1. There was no missing data. Seventeen (28%) patients had GERD as defined by ≥ 48 reflux episodes on 24-h esophageal pH-impedance reflux study. The median time from transplant to bronchoscopy, when LABW and BAL samples were concurrently obtained, was 3.02 months. The median follow-up time for CLAD-free survival was 4.67 years, for a total follow-up time of 260.59 person-years. The median follow-up time for overall survival was 5.04 years, for a total follow-up time of 288.66 person-years.

**Table 1:** Baseline patient characteristics

Characteristic	Patients (N = 61)
Median age (range)—years	59 (21–75)
Male sex—N (%)	35 (57)
Primary disease—N (%)	
Pulmonary fibrosis^a^	30 (49)
Chronic obstructive pulmonary disease	12 (20)
Cystic fibrosis	8 (12)
Other	11 (18)
GERD diagnosed by 24-h esophageal pH-impedance probe—N (%)	17 (28)
On proton pump inhibitor—N (%)	56 (92)
Median time from reflux testing to bronchoscopy (range)—days	11 (1–265)
Median time from transplant to bronchoscopy (range)—months	3.02 (1.81–3.95)
Acute rejection (A) grade—N (%)	
A0	36 (59)
A1 or higher	12 (20)
AX or no biopsy performed	13 (21)
Lymphocytic bronchiolitis (B) grade—N (%)	
B0	29 (48)
B1R	1 (2)
BX or no biopsy performed	31 (51)
Positive BAL microbiological culture^a^—N (%)	11 (18)
Donor recipient CMV status—N (%)	
Donor−/Recipient−	14 (23)
Donor−/Recipient +	15 (25)
Donor + /Recipient−	13 (21)
Donor + /Recipient +	19 (31)

*BAL* bronchoalveolar lavage, *CMV* cytomegalovirus, *GERD* gastroesophageal reflux

^a^One patient had a diagnosis of sarcoidosis. One had hypersensitivity pneumonitis. The rest (twenty-eight patients) had idiopathic pulmonary fibrosis

^b^Includes pathogenic bacteria, acid-fast bacilli, fungi, and viruses

### Biomarker levels in LABW versus BAL

**Fig. 2:**
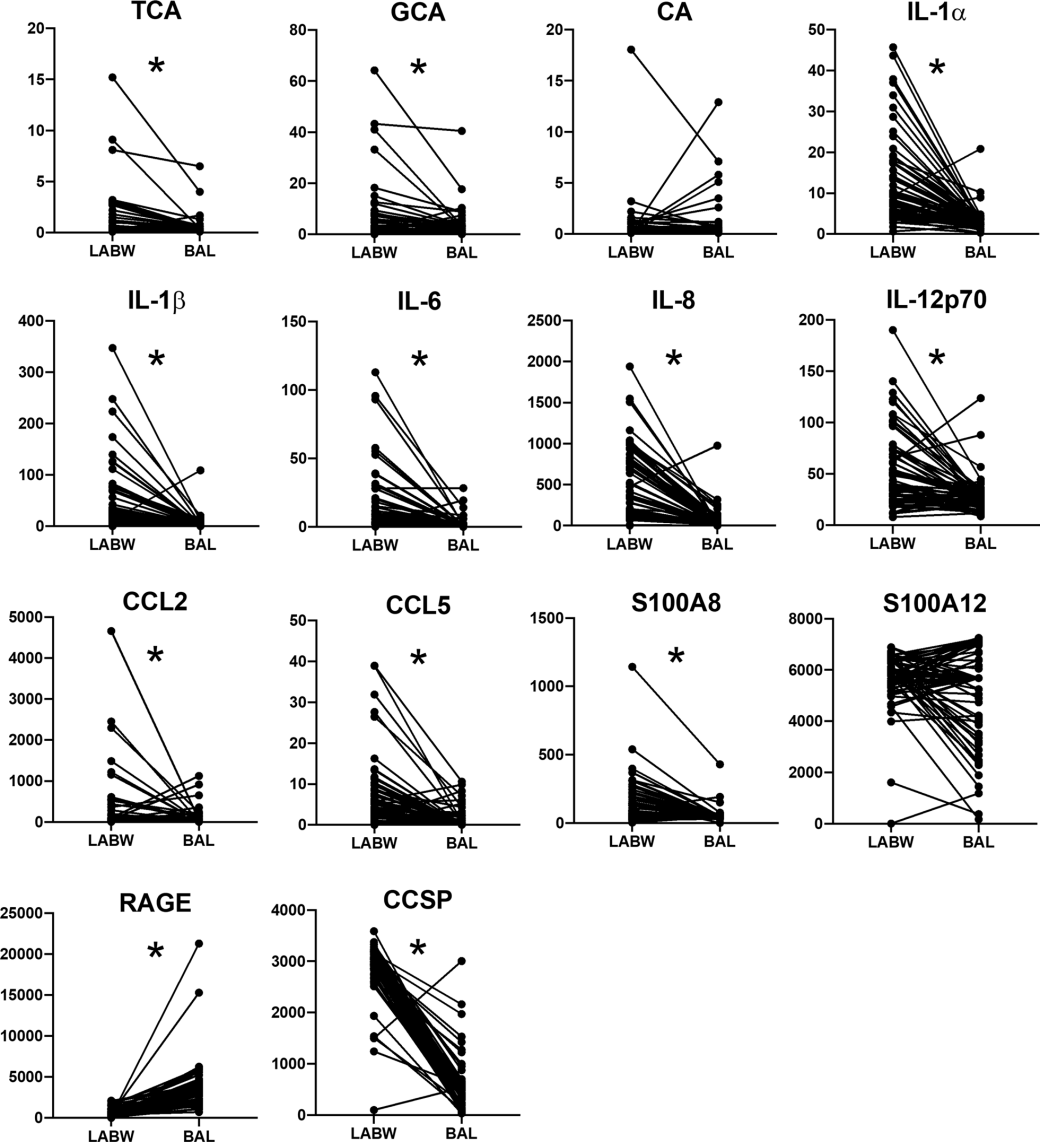
Biomarker levels in LABW vs. BAL. Wilcoxon signed rank test was used to compare levels of biomarkers in paired LABW vs BAL samples. Statistically significant differences as defined by *P* < 0.05 are indicated by a star. TCA, GCA, IL-1α, IL-1β, IL-6, IL-8, IL-12p70, CCL2, CCL5, S100A8, and CCSP were higher in LABW compared to BAL. RAGE was lower in LABW compared to BAL. CA and S100A12 levels were not statistically different between LABW and BAL. Y-axes units are nM for bile acids and pg/ml for proteins

The BAL biomarker levels were measured as part of our prior publication [16] and are included herein for purposes of comparison with LABW levels. Levels of TCA, GCA, IL-1α, IL-1β, IL-6, IL-8, IL-12p70, CCL2, CCL5, S100A8, and CCSP were higher in LABW compared to BAL (Fig. 2). RAGE levels were lower in LABW compared to BAL. CA and S100A12 levels were not statistically different between sample types.

### Biomarker correlations within and between samples

**Fig. 3:**
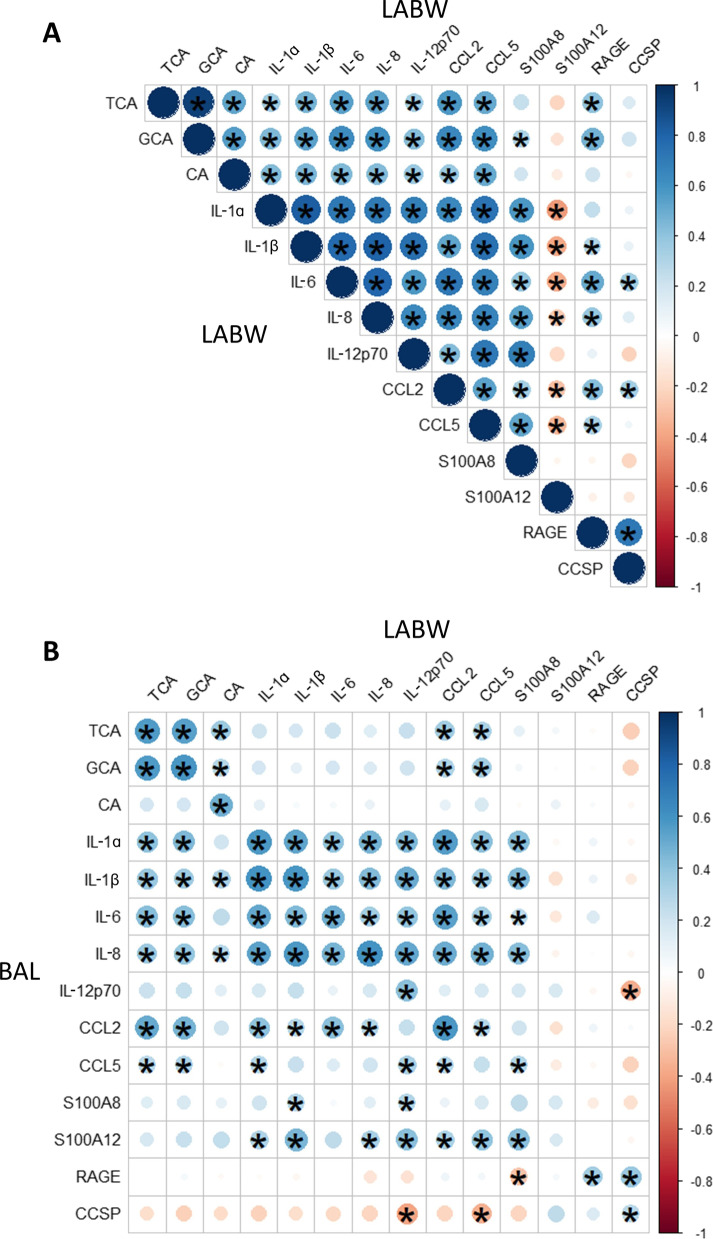
Heatmaps showing correlations between biomarker levels within LABW and between sample types. Spearman correlation was used to assess correlations between two biomarker levels within LABW (**A**) and between LABW and BAL (**B**). The colour and size of circles indicate respectively the direction (blue = positive; red = negative) and strength (larger = stronger; smaller = weaker) of correlation. Statistically significant correlations as defined by *P* < 0.05 are indicated by a star

Within LABW, nearly all bile acid and protein levels had significant positive correlations with each other, with the exception of S100A12 which was negatively correlated (Fig. 3A). Between sample types, LABW TCA and GCA positively correlated with the majority of BAL proteins, specifically IL-1α, IL-1β, IL-6, IL-8, CCL2, and CCL5 (Fig. 3B). BAL TCA and GCA positively correlated with only CCL2 and CCL5 in LABW.

### Association of biomarker levels with clinical outcomes

**Fig. 4:**
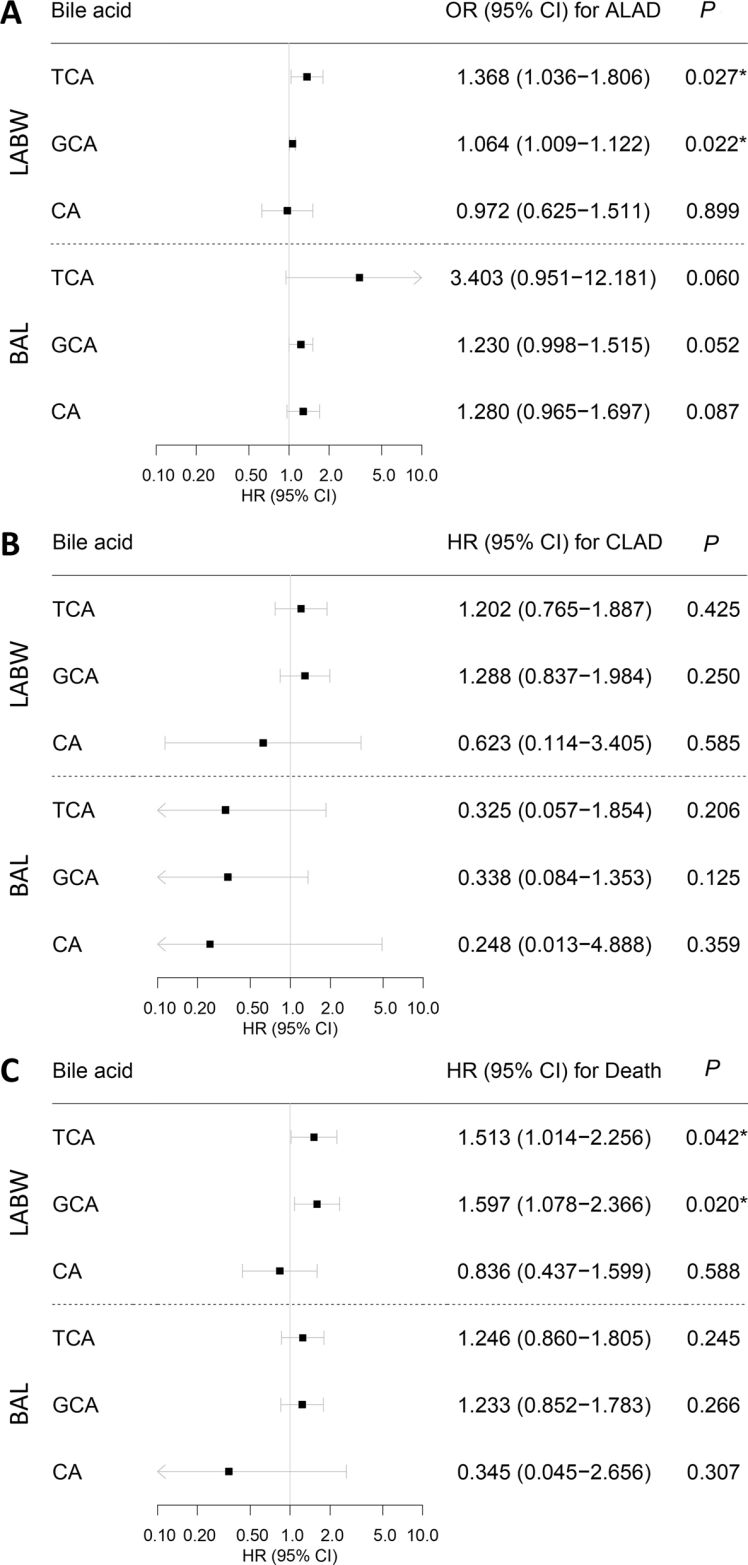
Forest plots showing association of bile acids with ALAD, CLAD, and death. Univariable logistic regression was used to assess associations of bile acid levels (as continuous variables) with ALAD (**A**). Multivariable Cox proportional hazards models, adjusted for recipient age, sex, primary disease, CMV mismatch, and concurrent acute rejection, were used to assess association of bile acid levels (as continuous variables) with time to CLAD (**B**) and time to death (**C**). Horizontal lines indicate 95% confidence intervals. Squares indicate point estimates. Statistically significant associations as defined by *P* < 0.05 are indicated by a star

Six (10%) patients had ALAD. LABW TCA and GCA were positively associated with ALAD in univariable logistic regression (OR = 1.368; 95%CI = 1.036–1.806; *P* = 0.027 and OR = 1.064; 95% CI = 1.009–1.122; *P* = 0.022, respectively). When GERD status was added to the model as a predictor, it was not significantly associated with ALAD, and the strength of associations between bile acids and ALAD remained unchanged (Additional file 1: Table S1). Bile acids in BAL were not significantly associated with ALAD (Fig. 4A).

No bile acids in LABW were associated with BLAD at up to 13 months (Additional file 1: Table S2). LABW TCA and GCA had weak but significant positive correlations with the number of proximal and total reflux episodes (Additional file 1: Fig. S1).

Sixteen (26%) patients developed CLAD, at a median follow-up time of 1.97 years. No bile acids in either LABW or BAL were significantly predictive of CLAD in multivariable Cox proportional hazards models adjusted for recipient age, sex, primary disease, CMV mismatch, and concurrent acute rejection (Fig. 4B).

Fifteen (25%) patients died, at a median follow-up time of 3.65 years. In multivariable Cox proportional hazards models, LABW TCA and GCA were independent predictors of death (HR = 1.513; 95% CI = 1.014–2.256; *P* = 0.042 and HR = 1.597; 95% CI = 1.078–2.366; *P* = 0.020, respectively). No bile acid in BAL significantly predicted death (Fig. 4C).

**Fig. 5:**
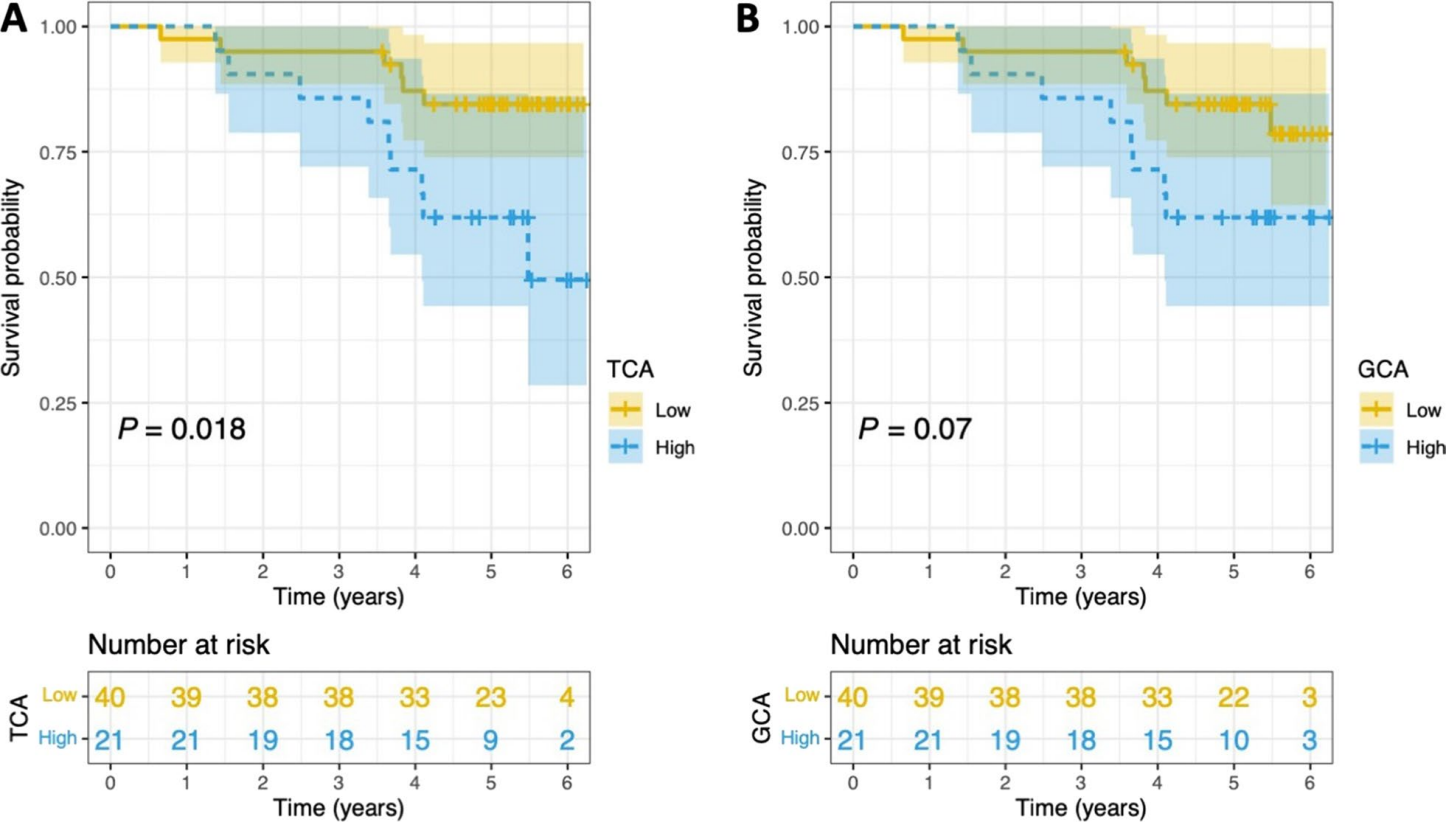
Kaplan–Meier curves for overall survival in patients stratified by TCA or GCA level. Kaplan–Meier survival curves with 95% confidence interval and number at risk table between patients in the highest tertile for TCA (**A**) or GCA (**B**) compared to all others. *P* value for log-rank test are shown

By Kaplan–Meier method, patients with LABW TCA in the top tertile (≥ 0.5 nM) had significantly worse overall survival compared to others (62% vs. 85% at 5 years; log-rank *P* = 0.018; Fig. 5A). Patients with LABW GCA in the top tertile (≥ 2.9 nM) had worse overall survival compared to others, which was not meeting the pre-specified level of significance (log-rank *P* = 0.07; Fig. 5B).

## Discussion

Our study reveals that bile acids, specifically TCA and GCA, are present at higher concentrations in LABW compared to BAL. This is consistent with the long-held theory that bile acid presence in the lungs is the result of microaspirated gastrointestinal contents [16]. Bile acid-containing refluxate initially enters the large airways and tracheobronchial tree, which are preferentially sampled by LABW. As refluxate travels more distally to small airways and alveolar space, it mixes with airway secretions and becomes progressively diluted, leading to the lower bile acid concentrations measured in BAL. We speculate this model is applicable in health and disease states, although impaired mucociliary clearance in lung transplant recipients may have exaggerated the effect in our study population [26].

In addition to bile acids, the vast majority of proteins are higher in LABW than in BAL. It is likely that variable dilution may play a role in our observed differences between LABW and BAL biomarker levels, since LABW involves instillation of 20 mL of isotonic saline as opposed to 50 mL for each BAL. However, the notable exception of RAGE being lower in LABW suggests that non-trivial reasons contribute, such as heterogeneous cell composition along the airways. Prior studies have shown RAGE is constitutively expressed by type 1 pneumocytes in the alveoli [27], which may account for its higher level in BAL. In contrast, CCSP is abundantly expressed within the conducting airway epithelium [28], explaining its higher level in LABW. In a study from another center where LABW was obtained prior to BAL (similar to our protocol), LABW recovered more epithelial cells and neutrophils, while BAL recovered more lymphocytes and alveolar macrophages [29]. Building upon these previous observations, our study further supports the idea that LABW and BAL can provide complementary information, as highlighted in the consensus guidelines for standardization of LABW collection and processing in lung transplantation. [2]

Similar to what we [16] and others [10] have reported in BAL, positive correlations exist between a majority of bile acids and inflammatory proteins in LABW. Our study examines this bile acid-inflammation link between sample types. We find that bile acids in LABW, specifically TCA and GCA, show strong positive correlations with many of the inflammatory proteins in BAL, specifically four proinflammatory cytokines and two chemokines. Conversely, TCA and GCA in BAL only correlate with two chemokines in LABW. The four BAL proinflammatory cytokines that correlate with LABW TCA and GCA (i.e. IL-1α, IL-1β, IL-6, IL-8) have all been implicated as key mediators of pulmonary inflammation and fibrosis. [30, 31]

LABW TCA and GCA are also the only bile acids in either sample type associated with a concurrent acute decline in lung function or ALAD. In our previous study on the larger BAL-only cohort of 76 patients, TCA and GCA were shown to be associated with ALAD [16]. After the exclusion of 15 patients who did not have matching LABW samples, BAL TCA and GCA do not demonstrate statistical significance in this present study, although both *P* values closely approach the statistical significance threshold. It is unclear whether lung inflammation and ALAD in our cohort were specifically due to microaspiration or other complications after lung transplantation such as acute rejection or infection. Nonetheless, LABW TCA and GCA appear to be more useful than their BAL counterparts in aiding the diagnosis of biologically and clinically relevant microaspiration in our cohort.

Consistent with the ALAD analysis, LABW TCA and GCA are the only bile acids in either sample type which are predictive of death after adjusting for major known risk factors. When stratifying our cohort by their bile acid levels (third tertile vs. first and second), TCA is better than GCA at identifying patients at higher risk of long-term mortality. These results are similar to those in our previous study, which found multiple strong signals with TCA, including associations with GERD, ALAD, and anti-reflux surgery. This was less so the case for GCA and not at all for CA. Overall, TCA and GCA seem more predictive than CA in our study. One of the important differences between these bile acid subspecies is that TCA and GCA are conjugated, whereas CA is unconjugated. Our findings confirm a recent study by Urso et al., which observed that conjugated bile acids in BW are associated with CLAD, mortality, and bacterial infections, in addition to changes in airway lipidome and cytokines [17]. As postulated by Urso et al., conjugated bile acids may be more deleterious to pulmonary epithelium compared to their unconjugated counterparts due to increased solubility. Our study thus provides additional evidence for the use of LABW over BAL in future mechanistic and translational research on bile acids.

Although our study focused on lung transplant recipients and microaspiration, it raises the question whether LABW sampling may be useful in general respiratory medicine. In contrast to previous studies that have compared the diagnostic yields of BAL and bronchial wash [3–5], ours is the first to identify a condition where LABW may offer superior clinical utility. Other possible conditions include tracheobronchial tuberculosis [32] or relapsing polychondritis [33], where the large airways and tracheobronchial space are preferentially affected. Moreover, there are certain clinical situations, such as in mechanically ventilated patients with acute respiratory distress syndrome, where significant hemodynamic instability, oxygen desaturation or even cardiac arrest have been reported after full volume BAL [34]. LABW may be considered a safer alternative to obtain an adequate sample for diagnostic purposes in these cases. We believe that future studies directly comparing between LABW and BAL in these broader clinical contexts would be helpful.

Our study’s strengths include the routine use of LABW and consistent bronchoscopy protocols at our lung transplant center. Given widely differing practices between institutions, with most centers rarely performing LABW, a multicenter study would be difficult. Another strength is our multimodal approach to analyzing samples, the completeness of follow-up, and the availability of clinical data which allowed us to evaluate relationships between biomarkers and relevant biological and clinical outcomes.

This work has several limitations which warrant discussion. Given its retrospective design, there are many potential unmeasured or unknown confounding variables which may have impacted the associations observed in our study. The study cohort was derived from a highly selected retrospective cohort of lung transplant recipients with and without GERD, approximately matched one to two. This limits the generalizability of our findings, as our study cohort may not be representative of the general lung transplant recipient population or patients with other respiratory diseases. Our sample size was limited, making it likely underpowered for multivariable survival analysis. Despite the low events and sample size in some analyses, we detected a consistent signal between LABW and BAL for TCA and GCA. However, our results should be interpreted with caution and require validation in larger studies before clinical translation.

## Conclusions

In this single-center retrospective cohort study, most bile acids and inflammatory proteins are present at higher levels in LABW compared to BAL at 3 months after lung transplantation. Compared to their counterparts in BAL, LABW TCA and GCA are more strongly associated with key inflammatory mediators and ALAD, and more predictive of death with adjustment of known risk factors. The clinical utility of LABW TCA and GCA as microaspiration biomarkers in lung transplant recipients require validation in a larger cohort. The potential of LABW as a sampling tool in research and clinical settings beyond lung transplantation should be further explored.

## Supplementary Information

**Additional file 1: Table S1.** Adjustment for GERD status in LABW bile acid and ALAD logistic regression. **Table S2.** Association of BLAD with LABW bile acids in univariable logistic regression. **Figure S1.** A: Spearman correlation of LABW TCA levels with reflux episodes. B: Spearman correlation of LABW GCA levels with reflux episodes. C: Spearman correlation of LABW CA levels with reflux episodes.

## Data Availability

The datasets used and/or analysed during the current study are available from the corresponding author on reasonable request.

## Abbreviations

ALAD: Acute lung allograft dysfunction
BA: Bile acid
BAL: Bronchoalveolar lavage
BLAD: Baseline lung allograft dysfunction
CA: Cholic acid
CCL2: C–C motif chemokine ligand 2
CCL5: C–C motif chemokine ligand 5
CCSP: Club cell secretory protein
CLAD: Chronic lung allograft dysfunction
ELISA: Enzyme-linked immunosorbent assay
FEV1: Forced expiratory volume in one second
GCA: Glycocholic acid
GERD: Gastroesophageal reflux disease
IL-12p70: Interleukin 12p70
IL-1α: Interleukin 1 alpha
IL-1β: Interleukin 1 beta
IL-6: Interleukin 6
IL-8: Interleukin 8
LABW: Large airway bronchial wash
RAGE: Receptor for advanced glycation endproducts
S100A12: S100 calcium-binding protein A12
S100A8: S100 calcium-binding protein A8
TCA: Taurocholic acid
