# Supplementary material for: Utility of bile acids in large airway bronchial wash versus bronchoalveolar lavage as biomarkers of microaspiration in lung transplant recipients: a retrospective cohort study
Source: Respir Res. 2022 Aug 26;23:219. doi: 10.1186/s12931-022-02131-5 (PMC9419323; doi:10.1186/s12931-022-02131-5)
Supplement: Supplementary file 1 — Additional file 1: Table S1. Adjustment for GERD status in LABW bile acid and ALAD logistic regression. Table S2. Association of BLAD with LABW bile acids in univariable logistic regression. Figure S1. A: Spearman correlation of LABW TCA levels with reflux episodes. B: Spearman correlation of LABW GCA levels with reflux episodes. C: Spearman correlation of LABW CA levels with reflux episodes. [file 12931_2022_2131_MOESM1_ESM.docx]

**Table S1: Adjustment for GERD status in LABW bile acid and ALAD logistic regression**

| **Model** | **Predictor** | **Odds ratio (95% Confidence Interval)** | **P value** |
| --- | --- | --- | --- |
| ALAD ~ TCA + GERD | TCA | 1.43 (1.09-1.89) | 0.01 |
|  | GERD | 0.15 (0.004-4.55) | 0.27 |
| ALAD ~ GCA + GERD | GCA | 1.07 (1.01-1.13) | 0.01 |
|  | GERD | 0.26 (0.16-4.27) | 0.34 |
| ALAD ~ CA + GERD | CA | 1.03 (0.64-1.48) | 0.89 |
|  | GERD | 2.06 (0.05-4.50) | 0.53 |

**Table S2: Association of BLAD with LABW bile acids in univariable logistic regression**

| **Predictor** | **Odds Ratio (95% Confidence Interval)** | **P value** |
| --- | --- | --- |
| TCA | 1.13 (0.89-1.42) | 0.29 |
| GCA | 1.03 (0.98-1.08) | 0.23 |
| CA | 0.56 (0.65-1.26) | 0.56 |

**Figure S1A: Spearman correlation of LABW TCA levels with reflux episodes**


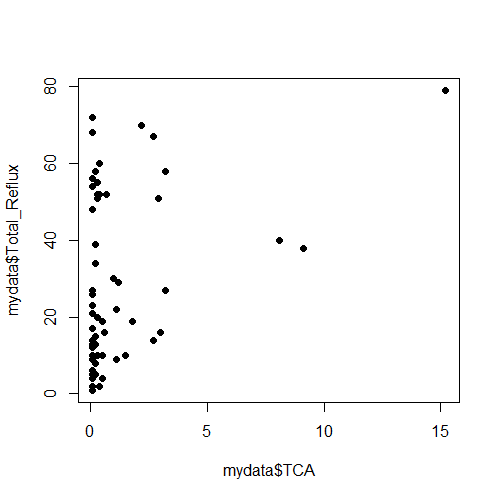


Total reflux episodes

TCA (nM)

r=0.31p=0.01


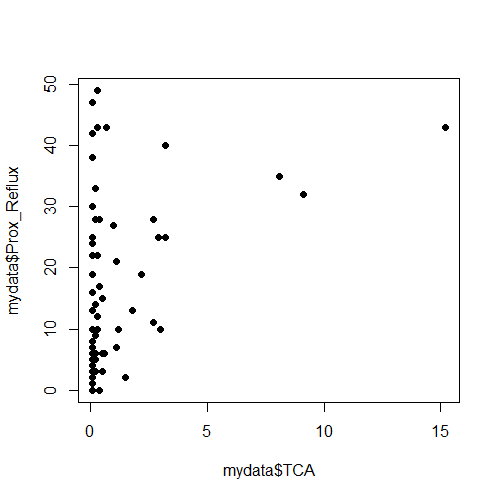


Proximal reflux episodes

TCA (nM)

r=0.29

p=0.02

**Figure S1B: Spearman correlation of LABW GCA levels with reflux episodes**


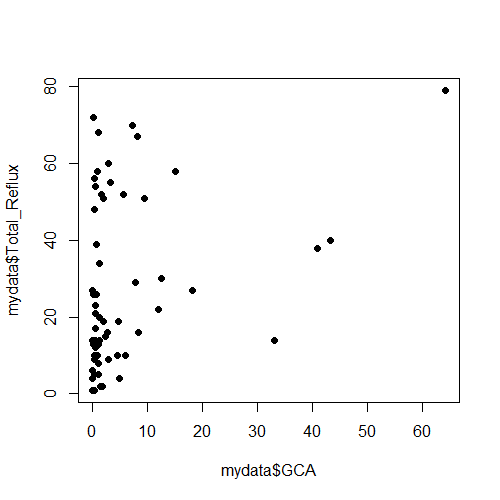


Total reflux episodes

GCA (nM)

r=0.33

p=0.01


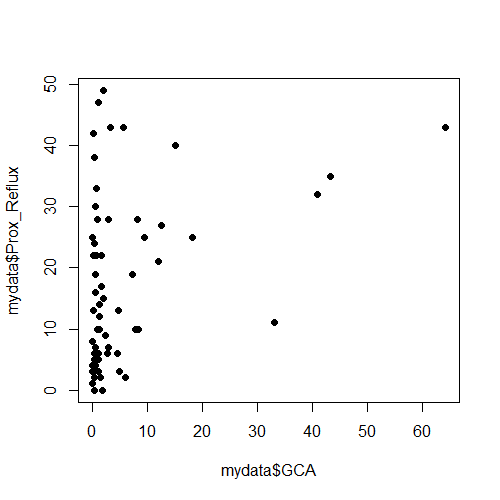


Proximal reflux episodes

GCA (nM)

r=0.32

p=0.01

**Figure S1C: Spearman correlation of LABW CA levels with reflux episodes**


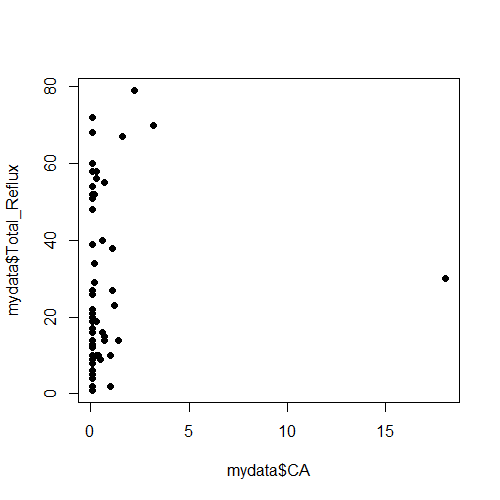


Total reflux episodes

CA (nM)

r=0.21

p=0.10


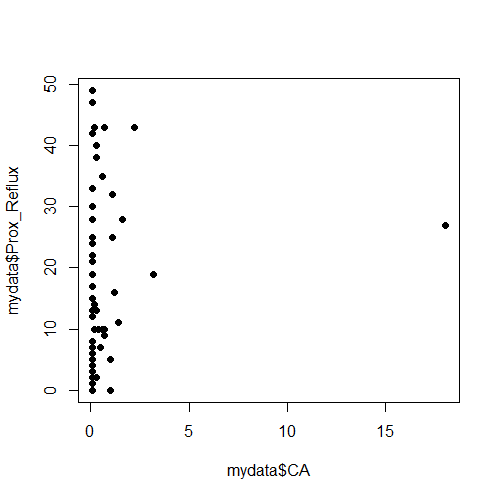


Proximal reflux episodes

CA (nM)

r=0.22

p=0.10
